# Thermal Effects on Tensile Behavior of Composite–Metal Hybrid Bolted Joints: Experimental and Numerical Study Based on Micromechanical Failure Theory

**DOI:** 10.3390/ma19132920

**Published:** 2026-07-07

**Authors:** Zixun Zhu, Rui Hou, Yue Liu, Wei Liu, Weicheng Gao

**Affiliations:** 1 Department of Astronautics Science and Mechanics, Harbin Institute of Technology, Harbin 150001, China; zzixun@hit.edu.cn (Z.Z.); rui_houh@126.com (R.H.); weiliu@hit.edu.cn (W.L.); 2 COMAC Shanghai Aircraft Design and Research Institute, Shanghai 200232, China; liuyue0018@126.com; 3 Department of Strength Design, The First Aircraft Institute of AVIC, Xi’an 710089, China

**Keywords:** composite–metal joints, thermal effects, multi-scale strategy, finite-element method, experimental validation

## Abstract

Accurately predicting the mechanical response and failure of composite–metal hybrid bolted joints under thermo-mechanical coupled loads remains a critical challenge in aerospace engineering. This paper develops a temperature-dependent multi-scale progressive failure analysis model based on micromechanical failure theory. A hexagonal representative volume element (RVE) incorporating fibers, matrix and interphase is constructed, with a stress amplification factor enabling macro–meso stress–strain transformation. Dimensionless temperature corrections are applied to resin and interphase mechanical properties, and temperature-influenced mesoscopic failure criteria with corresponding stiffness degradation schemes are proposed. The nonlinear progressive damage simulation is implemented via the ABAQUS/UMAT subroutine. Static tensile tests on AC531/CCF800H composite-7075 aluminum alloy three-bolt double-shear joints are conducted at −70 °C, 20 °C and 120 °C. The results show excellent agreement between the simulations and experiments, with ultimate load errors < 5%. Low temperature increases load capacity by 3.91% via resin hardening and enhanced interfacial bonding, while high temperature reduces it by 9.07% due to resin softening. Failure modes shift from end-hole tensile fracture (−70 °C, 20 °C) to full-hole bearing failure (120 °C), governed by altered bolt load distribution and damage evolution paths. The proposed model provides reliable support for thermo-mechanical design and strength verification of aerospace composite structures.

## 1. Introduction

Carbon fiber-reinforced polymer (CFRP) composites have been extensively applied in the primary load-bearing structures of aerospace and high-end equipment due to their superior specific strength, high specific modulus, and excellent designability [1]. Bolted connections between composite and metallic components represent the core assembly method in engineering systems. During service, these joints are frequently exposed to extreme thermal environments ranging from cryogenic to elevated temperatures. Temperature significantly alters the stress distribution, damage initiation, and ultimate load-bearing capacity of joint structures by modifying the resin matrix modulus, interfacial bonding state, and inducing thermal expansion mismatch between constituent materials [2]. According to the design specification of AC531/CCF800H composite laminates, the material is qualified for long-term service within the temperature range of −55 °C to 120 °C. Under specific aircraft mission operating conditions, the extreme operating temperature can reach −70 °C under high-altitude cruise conditions. Therefore, −70 °C (low temperature, LT), 20 °C (room temperature, RT), and 120 °C (high temperature, HT) were selected as test conditions to cover both the material design temperature range and the extreme service boundary of aircraft structures. Traditional macroscopic failure models struggle to reveal the mesoscopic failure mechanisms of fibers, matrix, and interphase under thermo-mechanical coupling. By contrast, micromechanics-based multiscale methods enable cross-scale failure prediction from constituent materials to structural levels, representing a critical approach to addressing this challenge.

Extensive experimental studies have been conducted on the room-temperature mechanical properties, bolt load distribution, and failure characteristics of composite bolted joints to elucidate the damage mechanisms and improve design standards. Previous research has primarily focused on the evolution of macroscopic CFRP properties under thermal environments, including tensile [3,4,5], compressive [6,7], and flexural properties [8,9]. Existing findings consistently demonstrate that matrix-related properties are highly temperature-dependent, whereas fiber mechanical properties remain relatively stable [10,11]. At low temperatures, the resin matrix undergoes brittle hardening with simultaneously enhanced interfacial bonding strength, leading to varying degrees of improvement in composite ultimate load capacity and initial stiffness [12]. At elevated temperatures, it is widely recognized that, when temperatures approach or exceed the resin glass transition temperature ($T_{g}$), the matrix transitions from a glassy to a rubbery state, resulting in a significant reduction in composite modulus and strength [13,14,15].

Regarding composite joint failure, Soykok et al. [16,17] evaluated the load-bearing capacity of bolted composite joints under low and high temperatures and compared their failure modes across the −40 °C to 80 °C range. The results showed that joints exhibited relatively higher load capacity at lower temperatures. Du et al. [18] conducted quasi-static tensile tests on carbon fiber-reinforced thermoplastic (CFRTP) joints from −25 °C to 100 °C. The peak tensile load decreased significantly with increasing temperature, with a maximum reduction of 51.18%. Failure was dominated by fracture at low and room temperatures but shifted to bearing failure at high temperatures. Song et al. [19] experimentally investigated carbon/epoxy single-lap riveted joints after thermal exposure, finding that specimen failure loads decreased by approximately 23–25%. This severe capacity degradation was attributed to high-temperature-induced thermal damage to the matrix.

Due to the inherent property dispersion of composites and complex experimental conditions, conducting extensive tests to characterize the mechanical performance of composites and their joints under thermal environments is both costly and labor-intensive. Therefore, reliable and efficient numerical methods for composite failure prediction are urgently needed in structural design and analysis. In multi-scale analysis, researchers have characterized fiber and matrix failure modes separately at the mesoscale using micromechanics-based stress transformation methods for representative unit cells (RUCs) and established the corresponding failure criteria and damage evolution models [20,21,22,23]. Hu et al. [24,25] developed a temperature-dependent anisotropic continuum damage model based on continuum damage mechanics to simulate the damage and failure behavior of composite joints during interference fit, bolt preloading, thermal loading, and mechanical loading. Zhao et al. [26] established a composite degradation model based on the representative volume element (RVE), which accurately predicted typical failure modes of bolted joints, including tension, shear, splitting, and bearing, providing a micromechanical foundation for progressive damage analysis.

While substantial experimental and numerical efforts have been devoted to composite bolted joints at room temperature, multi-scale mesoscopic failure prediction of hybrid composite–metal joints under thermo-mechanical coupling remains a critical unresolved challenge. Traditional macroscopic models cannot capture the distinct failure behaviors of individual constituents under combined thermal and mechanical loads, whereas micromechanics-based multi-scale approaches offer a promising pathway for cross-scale failure prediction.

Against this background, this study focuses on three-bolt double-shear joints composed of AC531/CCF800H composite and 7075 aluminum alloy. A temperature-dependent multi-scale progressive failure model is developed based on micromechanical theory. A mesoscopic RVE model incorporating fibers, matrix, and interphase is constructed, and temperature-dependent material constitutive relations and macro–meso stress transformation are established. Thermally coupled mesoscopic failure criteria and corresponding stiffness degradation schemes are proposed. A UMAT subroutine is implemented in ABAQUS to perform nonlinear progressive damage simulations, and static tensile tests are conducted at −70 °C (low temperature, LT), 20 °C (room temperature, RT), and 120 °C (high temperature, HT) for model validation. The effects of temperature on ultimate load capacity, load-displacement response, bolt load distribution, and damage evolution are systematically analyzed to reveal the underlying mechanisms governing failure mode transitions. This work provides theoretical support for the strength design and engineering application of composite–metal hybrid joints in extreme thermal environments.

## 2. Progressive Failure Analysis Model Considering Thermal Effect

A multi-scale analytical model is proposed to predict the failure behavior of composite structures subjected to combined thermal and mechanical loads. Mesoscopically, the model accounts for temperature effects on stress analysis, failure criteria and material degradation models of carbon fibers, resin matrix and interphase. A detailed flowchart is presented, and the implementation procedure of the model is elaborated.

### 2.1. Modelling of Representative Volume Element

CFRP composites are arranged randomly yet exhibit statistically periodic distribution characteristics. Accordingly, the RVE is established based on the periodicity assumption. The overall composite structure can be regarded as an assembly of periodically arranged RVEs. Under remote uniform loading, all RVEs present periodic stress and strain responses. Hence, the mechanical responses of the entire composite can be effectively evaluated by analyzing a single RVE, and the macroscopic performance and structural response can be further derived.

**Figure 1:**
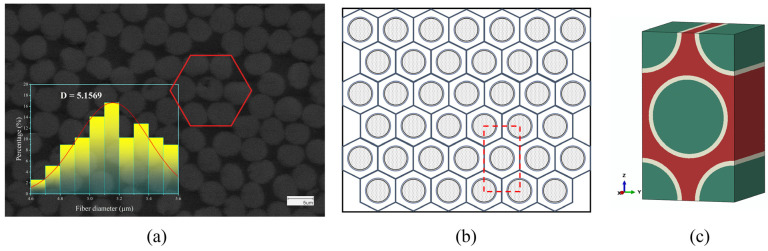
Scanning electron microscopy (SEM) observation of composite cross-section and hexagonal representative volume element model: (**a**) SEM image and fiber diameter statistics; (**b**) hexagonal fiber distribution assumption; (**c**) hexagonal RVE.

Scanning electron microscope observation is conducted to capture the microscopic morphology of AC531/CCF800H carbon fiber-reinforced epoxy composites (AVIC Composite Corporation Ltd., Beijing, China). The fiber volume fraction $V_{f}$ is determined as 57%, with a fiber diameter of 5.16 μm and an interphase thickness of 0.5 μm, as illustrated in Figure 1. An interphase thickness of 0.5 μm is adopted as a typical modeling assumption for the three-phase RVE, which lies within the commonly reported range (0.2–1 μm) for carbon fiber–epoxy composite systems in published micromechanical studies [27].

Numerous studies demonstrate that the hexagonal fiber distribution assumption can well-simulate the practical fiber arrangement within composites [20,28,29]. Therefore, this study adopts the hexagonal distribution hypothesis. Taking the distance between opposite sides of the hexagonal RVE as the unit length, the finite-element model of the microscopic representative volume element is established, as shown in Figure 1c.

### 2.2. Conversion of Macro-Mesoscopic Stress Accounting for Thermal Effects

CFRP composites are susceptible to thermal loads. Due to material anisotropy, obvious dimensional and morphological variations occur along three principal material directions. For anisotropic composites under thermal environments, the strain–stress relation is established by combining thermal strain and stress-induced strain in the principal material coordinate system:

(1) $$\bar{\sigma }\;=\;\bar{C}\times \left(\bar{\varepsilon }-\bar{\alpha }\Delta T\right)$$

where $\bar{\sigma }$, $\bar{\varepsilon }$ denote the macroscopic stress and strain, respectively; $\bar{C}$ represents the macroscopic stiffness matrix of the material; and ΔT is the temperature difference between the RT and the current temperature.

For orthotropic materials, Equation (1) can be rewritten in component form as:

(2) $$\left(\begin{array}{c} {\bar{\sigma }}_{1}\\ {\bar{\sigma }}_{2}\\ {\bar{\sigma }}_{3}\\ {\bar{\sigma }}_{4}\\ {\bar{\sigma }}_{5}\\ {\bar{\sigma }}_{6} \end{array}\right)=\left[\begin{array}{cccccc} {\bar{C}}_{11} & {\bar{C}}_{12} & {\bar{C}}_{13} & 0 & 0 & 0\\ {\bar{C}}_{12} & {\bar{C}}_{22} & {\bar{C}}_{23} & 0 & 0 & 0\\ {\bar{C}}_{13} & {\bar{C}}_{23} & {\bar{C}}_{33} & 0 & 0 & 0\\ 0 & 0 & 0 & {\bar{C}}_{44} & 0 & 0\\ 0 & 0 & 0 & 0 & {\bar{C}}_{55} & 0\\ 0 & 0 & 0 & 0 & 0 & {\bar{C}}_{66} \end{array}\right]\left(\begin{array}{c} {\bar{\varepsilon }}_{1}-{\bar{\alpha }}_{1}\Delta T\\ {\bar{\varepsilon }}_{2}-{\bar{\alpha }}_{2}\Delta T\\ {\bar{\varepsilon }}_{3}-{\bar{\alpha }}_{3}\Delta T\\ {\bar{\varepsilon }}_{4}\\ {\bar{\varepsilon }}_{5}\\ {\bar{\varepsilon }}_{6} \end{array}\right)$$

The mechanical properties of unidirectional laminates are governed by the characteristics of fibers, matrix and interphase. Macroscopic stresses are converted into mesoscopic stresses at reference points of the representative volume element via stress amplification factors, which enables efficient acquisition of corresponding mesoscopic stress values. The temperature-dependent evolution formulas of mesoscopic and macroscopic stresses are expressed as follows:

(3) $$\sigma \;=\;M^{SAF}\bar{\sigma }+A^{SAF}\Delta T$$

where σ is the mesoscopic stress; $M^{SAF}$ is the stress amplification factor for macroscopic stress; and $A^{SAF}$ is the stress amplification factor for thermal stress.

Expanding on $M^{SAF}$, the conversion between macroscopic stress and microscopic stress at the reference point can be further expressed as:

(4) $$\left\{\begin{array}{c} \sigma_{11}\\ \sigma_{22}\\ \sigma_{33}\\ \tau_{12}\\ \tau_{13}\\ \tau_{23} \end{array}\right\}={\left[\begin{array}{cccccc} M_{11} & M_{12} & M_{13} & M_{14} & M_{15} & M_{16}\\ M_{21} & M_{22} & M_{23} & M_{24} & M_{25} & M_{26}\\ M_{31} & M_{32} & M_{33} & M_{34} & M_{35} & M_{36}\\ M_{41} & M_{42} & M_{43} & M_{44} & M_{45} & M_{46}\\ M_{51} & M_{52} & M_{53} & M_{54} & M_{55} & M_{56}\\ M_{61} & M_{62} & M_{63} & M_{64} & M_{65} & M_{66} \end{array}\right]}^{SAF}\left\{\begin{array}{c} {\bar{\sigma }}_{11}\\ {\bar{\sigma }}_{22}\\ {\bar{\sigma }}_{33}\\ {\bar{\tau }}_{12}\\ {\bar{\tau }}_{13}\\ {\bar{\tau }}_{23} \end{array}\right\}+{\left[\begin{array}{c} A_{11}\\ A_{21}\\ A_{31}\\ A_{41}\\ A_{51}\\ A_{61} \end{array}\right]}^{SAF}\Delta T$$

**Figure 2:**
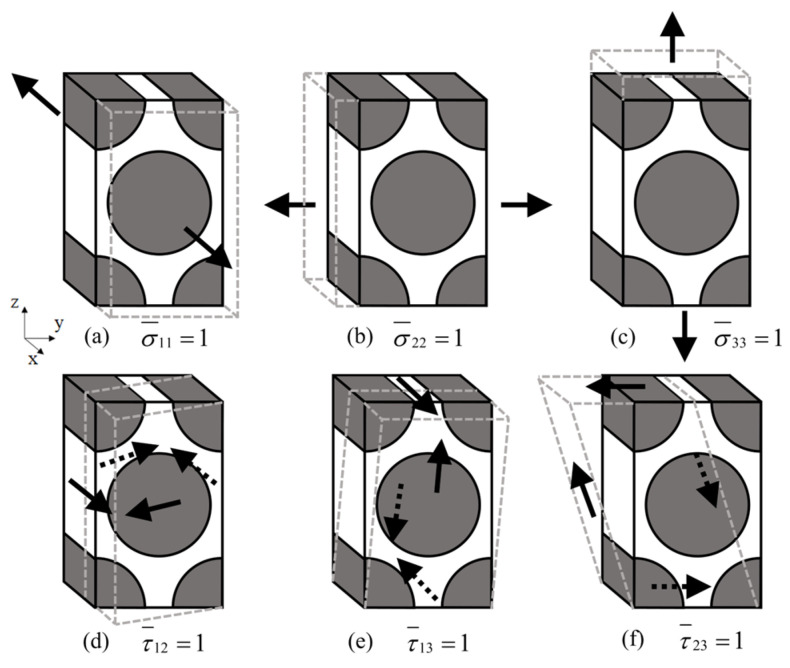
Unit stresses applied on the micro-scale RVE: (**a**) RVE under ${\bar{\sigma }}_{11}=1$; (**b**) RVE under ${\bar{\sigma }}_{22}=1$; (**c**) RVE under ${\bar{\sigma }}_{33}=1$; (**d**) RVE under ${\bar{\tau }}_{12}=1$; (**e**) RVE under ${\bar{\tau }}_{13}=1$; (**f**) RVE under ${\bar{\tau }}_{23}=1$.

Stress amplification factors can be determined using RVE. The stress amplification factors are calculated based on the periodic RVE model under unit load conditions, with the complete implementation process as follows:

- First, periodic boundary conditions (PBCs) are applied to all six surfaces of the hexagonal RVE via linear constraint equations in ABAQUS. This ensures the displacement and stress fields of the RVE satisfy periodic continuity, which is consistent with the assumption of statistically uniform fiber distribution in composites;
- For mechanical stress amplification factors, six independent loading cases are conducted on the RVE: a unit macroscopic normal stress (${\bar{\sigma }}_{11}=1$, ${\bar{\sigma }}_{22}=1$, ${\bar{\sigma }}_{33}=1$) or unit macroscopic shear stress (${\bar{\tau }}_{12}\;=\;1$, ${\bar{\tau }}_{13}=1$, ${\bar{\tau }}_{23}=1$) is applied, respectively, while all other stress components are set to zero, as shown in Figure 2. After finite-element solving, the full mesoscopic stress tensor at each reference point is extracted. By substituting the extracted mesoscopic stress tensors into Equation (4), the mechanical stress amplification factor matrix $M^{SAF}$ can be obtained;
- For thermal stress amplification factors, an additional loading case with a unit temperature increment (Δ*T* = 1 K) is applied to the RVE with all mechanical loads removed. The thermal-induced mesoscopic stress at each reference point is extracted, and the thermal stress amplification factor vector $A^{SAF}$ is defined as: $A_{ij}=\sigma_{ij}/\Delta T$.

It should be noted that both the $M^{SAF}$ and $A^{SAF}$ matrices are recalculated independently for each service temperature. Since the elastic and thermal properties of the matrix and interphase vary with temperature, the stiffness field and thermal deformation field inside the RVE differ across temperature conditions. Separate recalculation of SAF matrices at each target temperature guarantees the accuracy of the macro–meso stress transformation throughout the analysis. Mechanical and thermal stress amplification factors are acquired by extracting microscopic stress components at designated reference points.

**Figure 3:**
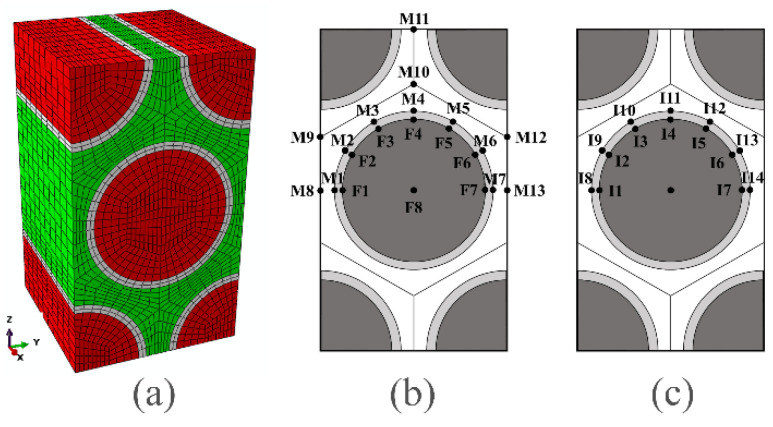
Micro-scale RVE of three-phase materials: (**a**) 3D finite element mesh of the three-phase RVE; (**b**) Sampling reference points distributed on fiber and matrix regions (F: fiber surface points, M: matrix boundary points); (**c**) Sampling reference points distributed on the fiber-matrix interphase layer (I: interphase boundary points).

The finite-element model of interphase-included RVE and selection of reference points for stress amplification factor calculation are presented in Figure 3. Figure 3a shows the established RVE finite-element model, where thirty-five reference points are selected covering maximum stress locations under six stress states and other critical regions. The selection of reference points strictly follows the classic micromechanical failure analysis framework proposed by Ha et al. [22,23] and validated in our previous work [20,21]. All reference points are arranged at typical stress concentration positions within the RVE, which fully cover all potential damage initiation locations of fiber, matrix and interphase constituents. The specific distribution rules are as follows:

- Fiber region (8 points, F1–F8): Points are placed at the fiber–interphase interface along transverse and shear loading directions, as well as the fiber center and axial stress extremum positions. These points cover the high-stress zones of fiber under axial tension/compression and transverse loading, which dominate fiber failure initiation, as shown in Figure 3b;
- Matrix region (13 points, M1–M13): Points are distributed in the inter-fiber matrix bridge, the matrix region near the fiber gap, and the matrix adjacent to the interphase. These positions correspond to the peak regions of matrix tensile stress, compressive stress and shear stress, where matrix cracking usually initiates first;
- Interphase region (14 points, I1–I14): Points are uniformly selected along the circumferential direction of the fiber–matrix interface, including typical angular positions (0°, 45°, 90°, 135°, etc.) where interfacial normal stress and shear stress reach their peaks under different loading directions. This arrangement ensures the capture of interfacial debonding initiation under arbitrary combined stress states, as illustrated in Figure 3c.

The mechanical and thermal stress amplification factors $M^{SAF}$ and $A^{SAF}$ in Equation (4) can be obtained at each reference point, supporting subsequent failure judgment of fiber, matrix and interphase constituents.

### 2.3. Material Properties Accounting for Thermal Effects

Carbon fibers exhibit stable material properties under thermal environments. The elastic modulus, strength, and coefficient of thermal expansion (CTE) of CCF800H carbon fibers are presented in Table 1; these parameters are less affected by temperature and are generally considered temperature-independent. The material properties of AC531 epoxy resin at RT are shown in Table 2.

Unlike carbon fibers, the material properties of epoxy resin are significantly affected by temperature, and its stiffness degradation with increasing temperature exhibits nonlinear accelerating characteristics. The dimensionless temperature $T^{*}$ proposed by Tsai [30] is a fundamental parameter for evaluating the performance of composites under thermal effects, which is defined as:

(5) $$T^{*}=\frac{T_{g}-T}{T_{g}-T_{0}}$$

where $T_{g}$ and $T_{0}$ represent the glass transition temperature and RT, respectively, and T denotes the service temperature.

When the temperature approaches $T_{g}$, the resin matrix transforms into a high-elastic state, accompanied by a sharp modulus reduction of 60–80%. The elastic modulus is assumed to decline to zero at this critical temperature. Based on Tsai’s equation, the temperature-dependent elastic modulus can be expressed by the dimensionless temperature $T^{*}$ as:

(6) $$\frac{E_{m}^{T}}{E_{m}}={\left(T^{*}\right)}^{a}$$

where $E_{m}$ and $E_{m}^{T}$ denote the elastic moduli of AC531 resin at RT and at temperature T, respectively. Parameter a is the power-law exponent determined by the material.

For the AC531 epoxy resin adopted in this study, the glass transition temperature $T_{g}$ is 230 °C. The maximum test temperature of 120 °C is far below $T_{g}$, where the resin matrix remains in the stable glassy state without glass–rubber phase transition. The mechanical properties (elastic modulus and strength) degrade gradually and continuously with increasing temperature in this interval. Therefore, the temperature-dependent constitutive model based on the dimensionless temperature power law exhibits sufficient accuracy and applicability within the studied temperature range.

**Figure 4:**
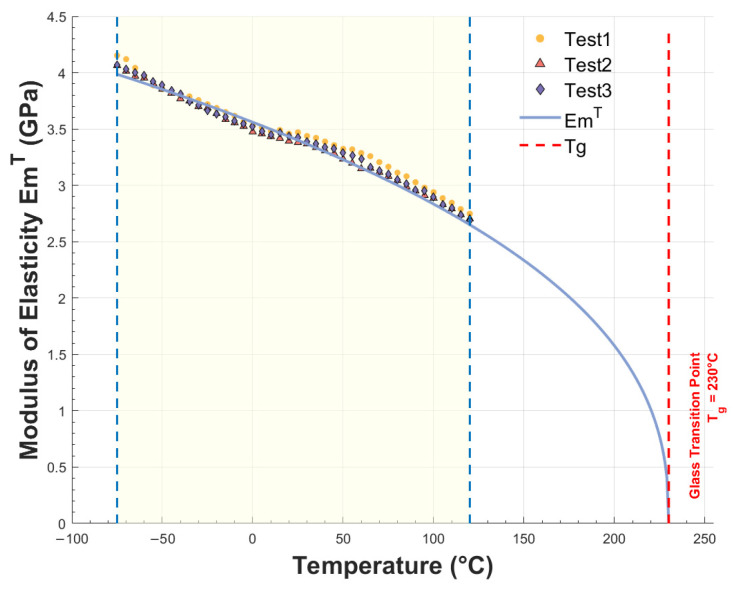
Elastic modulus of AC531 resin vs. temperature.

To determine parameter a in the power-law model, tensile tests were performed at 5 °C intervals covering the temperature range from LT to HT following ASTM D638-14 [31] Standard Test Method for Tensile Properties of Plastics. Three parallel specimens were measured at each temperature point to guarantee data reliability. The relationship between modulus and temperature is shown in Figure 4, and parameter a = 0.4 was obtained using the nonlinear least squares method for fitting, with a coefficient of determination R^2^ = 0.9787 reflecting high fitting precision between the model curve and the experimental measurements. The coefficient of variation of the elastic modulus at all test temperatures is lower than 2.8%, which demonstrates small data dispersion and excellent repeatability of the tensile experiments.

The Poisson’s ratio $v_{m}$ of the resin matrix is less affected by temperature and is treated as a constant. The CTE $\alpha_{m}$ increases continuously with an increasing temperature. For the convenience of calculation, the engineering CTE at temperature T is taken with $T_{0}$ as the reference point.

Furthermore, temperature exerts a remarkable influence on the strength of the resin matrix. The correlation between temperature and strength is obtained by fitting the experimental data [32].

(7) $$\frac{X_{m}^{T}}{X_{m}}={\left(T^{*}\right)}^{0.2}$$

where $X_{m}$ denotes the strength of the resin matrix at RT, and $X_{m}^{T}$ denotes the strength of the resin matrix at temperature T.

In CFRP composites, the thickness of the fiber–matrix interphase is generally extremely thin, making its modulus distribution difficult to measure experimentally. Anifantis [33] proposed an assumption that the elastic modulus, Poisson’s ratio and thermal expansion coefficient vary continuously within the interphase. The fiber–matrix interphase shares similar mechanical characteristics with the resin matrix and conforms to the isotropic hypothesis. The tensile modulus, Poisson’s ratio and thermal expansion coefficient of the interphase can be expressed as functions of the radial coordinate *r* in cylindrical coordinates:

(8) $$\left\{\begin{array}{l} E_{int}(r)=E_{m}\left[1+p^{E}\frac{E_{2,f}-E_{m}}{E_{m}}\frac{1-re^{1-r/(r_{f}+d_{int})}/(r_{f}+d_{int})}{1-r_{f}e^{1-r_{f}/(r_{f}+d_{int})}/(r_{f}+d_{int})}\right]\\ v_{int}(r)=v_{m}\left[1+p^{v}\frac{v_{12,f}-v_{m}}{v_{m}}\frac{1-re^{1-r/(r_{f}+d_{int})}/(r_{f}+d_{int})}{1-r_{f}e^{1-r_{f}/(r_{f}+d_{int})}/(r_{f}+d_{int})}\right]\\ \alpha_{int}(r)=\alpha_{m}\left[1+p^{\alpha }\frac{\alpha_{22,f}-\alpha_{m}}{\alpha_{m}}\frac{1-re^{1-r/(r_{f}+d_{int})}/r(r_{f}+d_{int})}{1-r_{f}e^{1-r_{f}/(r_{f}+d_{int})}/r(r_{f}+d_{int})}\right] \end{array}\right.$$

where *r* is the radial component in cylindrical coordinates; $r_{f}$ is the fiber radius; and $d_{int}$ is the interfacial phase thickness. $p^{E}$, $p^{v}$, and $p^{\alpha }$ are dimensionless adhesion efficiency factors, serving to quantify the mechanical properties between the interfacial layer and the fiber. By adjusting the adhesion efficiency factors, the model can simulate the effect of interfacial defects (e.g., debonding and cracks) on the mechanical behavior of composites.

The dimensionless adhesion efficiency factor ranges from zero to one. A value of one represents perfect, defect-free fiber–interphase bonding with full stress transfer, while a value approaching zero corresponds to complete interfacial debonding with nearly zero load transmission. For the studied AC531/CCF800H unidirectional CFRP composite, tiny microcavities and partial weak bonding inevitably exist at the fiber–resin interphase during autoclave curing molding, leading to incomplete stress transfer between the fiber and the matrix. According to reference [34], the adhesion efficiency factors $p^{E}$, $p^{v}$ and $p^{\alpha }$ are taken as 0.6.

Interfacial stiffness properties are defined as the ratio of the definite integral of stiffness properties over the interfacial thickness to the interfacial thickness:

(9) $$\left\{\begin{array}{l} {\bar{E}}_{int}=\int_{r_{f}}^{r_{f}+d_{int}}E_{int}(r)dr/d_{int}\\ {\bar{v}}_{int}=\int_{r_{f}}^{r_{f}+d_{int}}v_{int}(r)dr/d_{int}\\ {\bar{\alpha }}_{int}=\int_{r_{f}}^{r_{f}+d_{int}}\alpha_{int}(r)dr/d_{int} \end{array}\right.$$

where ${\bar{E}}_{int}$ denotes the mean value of the interfacial phase’s elastic modulus, ${\bar{\nu }}_{int}$ denotes the mean value of the interfacial phase’s Poisson’s ratio, and ${\bar{\alpha }}_{int}$ denotes the mean value of the interfacial phase’s CTE.

Substituting the transverse properties of CCF800H carbon fiber from Table 1 and the AC531 epoxy matrix properties from Table 2 into Equations (8) and (9), the equivalent average properties of the interphase at the reference temperature (20 °C) can be obtained analytically. The calculated elastic modulus ${\bar{E}}_{int}$, Poisson’s ratio ${\bar{v}}_{int}$ and coefficient of thermal expansion ${\bar{\alpha }}_{int}$ of the interphase are 8.15 GPa, 0.291, and 26.55 × 10^−6^ K^−1^, respectively. For the strength parameters of the interphase, the reference normal tensile strength $X_{n,int}$ and interfacial shear strength $X_{t,int}$ at 20 °C are determined via micro-mechanical experimental characterization of the fiber–matrix interface. The $X_{n,int}$ and the $X_{t,int}$ of the fiber-matrix interface are 60 MPa and 45 MPa, respectively.

Similar to the resin matrix, the elastic modulus and strength of the interlaminar phase at temperature T can be expressed in terms of the dimensionless temperature $T^{*}$ as:

(10) $$\frac{{\bar{E}}_{int}^{T}}{{\bar{E}}_{int}}={\left(T^{*}\right)}^{0.4},\;\frac{X_{int}^{T}}{X_{int}}={\left(T^{*}\right)}^{0.2}$$

where $X_{int}$ denotes the strength of the interlaminar phase at RT, and $X_{int}^{T}$ denotes the strength of the interfacial phase at temperature T.

### 2.4. Mesoscopic Failure Criteria and Degradation Model Considering Thermal Effects

In CFRP composites, they can be divided into three constituent regions—fiber, matrix, and interfacial phase—based on differences in properties, and their failures can be categorized into fiber failure, matrix failure, and fiber–matrix interfacial debonding.

#### 2.4.1. Fiber Failure

Reinforcing fibers show obvious orthotropic characteristics. Their strengths and stiffnesses are much higher than those of the matrix and interphase. When the composite is loaded along the 0° direction, fibers bear most of the load. Thus, failure in this direction is dominated by fibers. Fibers are brittle, and their fracture occurs perpendicular to the fiber orientation. The maximum stress criterion is adopted to judge fiber failure, which is expressed as:

(11) $$\begin{array}{c} \sigma_{1,f}\geq X_{t,f}\\ \sigma_{1,f}\leq -X_{c,f} \end{array}$$

where $X_{t,f}$ denotes the tensile strength of the fiber, and $X_{c,f}$ denotes its compressive strength.

#### 2.4.2. Matrix Failure

Epoxy matrix in composites is generally isotropic. Many tests show that its tensile strength differs from its compressive strength. Defects such as microcracks and voids exist inside the matrix. The matrix is more sensitive to tensile stress than compressive stress when failure occurs. The difference between tensile and compressive strength indicates that damage initiation of the matrix depends on deviatoric stress invariants (e.g., von Mises stress, $\sigma_{VM,m}$) and volumetric stress invariants (e.g., the first stress invariant $I_{1}$). The first stress invariant, second stress invariant and stress expressions for the matrix at the mesoscale are given below:

(12) $$I_{1}=\sigma_{11,m}+\sigma_{22,m}+\sigma_{33,m}$$

(13) $$I_{2}=\sigma_{11,m}\sigma_{22,m}+\sigma_{22,m}\sigma_{33,m}+\sigma_{11,m}\sigma_{33,m}-({\tau_{12,m}}^{2}+{\tau_{13,m}}^{2}+{\tau_{23,m}}^{2})$$

(14) $$\sigma_{VM,m}=\sqrt{{I_{1}}^{2}-3I_{2}}$$

To account for the effects of both deviatoric stress invariants and volumetric stress invariants on matrix failure simultaneously, Bauwens [35] and Raghava [36], et al. proposed a modified criterion based on the Mises criterion to determine matrix damage initiation:

(15) $$\frac{{\sigma_{VM,m}}^{2}}{X_{t,m}^{T}X_{c,m}^{T}}+\left(\frac{1}{X_{t,m}^{T}}-\frac{1}{X_{c,m}^{T}}\right)I_{1,m}=1$$

where $X_{t,m}^{T}$ denotes the tensile strength of the matrix at operating temperature T, and $X_{c,m}^{T}$ denotes its compressive strength at temperature T.

#### 2.4.3. Interphase Failure

Under the combined influence of thermal and mechanical loading, interfacial debonding occurs due to the mismatch in the CTE between the fiber and the matrix, leading to the superposition of thermal and mechanical stresses. Therefore, in addition to matrix failure and fiber failure, interfacial debonding between the fiber and the resin matrix is also a primary failure mode. This study adopts the interfacial damage initiation criterion modified by Ye [37] based on the Hashin–Rotem criterion:

(16) $$\left\{\begin{array}{l} {\left(\frac{{\bar{\sigma }}_{int}}{X_{n,int}^{T}}\right)}^{2}+{\left(\frac{{\bar{\tau }}_{int}}{X_{t,int}^{T}}\right)}^{2}=1\;\;\;\;({\bar{\sigma }}_{int}>0)\\ {\left(\frac{{\bar{\tau }}_{int}}{X_{t,int}^{T}}\right)}^{2}=1\;\;\;\;\;\;\;\;\;\;\;\;\;\;\;\;\;\;\;\;\;\;\;\;\;\;({\bar{\sigma }}_{int}\leq 0) \end{array}\right.$$

where ${\bar{\sigma }}_{int}$ and ${\bar{\tau }}_{int}$ represent the interfacial normal stress and interfacial shear stress, respectively; $X_{n,int}^{T}$ and $X_{t,int}^{T}$ denote the interfacial normal tensile strength and interfacial shear strength of the interphase at service temperature T.

#### 2.4.4. Stiffness Degradation

When the mesoscopic stress field of fibers, matrix, or interphase in composites satisfies any of the above failure criteria, the corresponding damage failure mode is considered to have occurred in that constituent material. As damage develops in composites, the mechanical properties of the damaged material degrade, which manifests as a reduction in the macroscopic equivalent stiffness of the material. At this point, appropriate stiffness degradation must be applied to the material. In thermal environments, the coefficients in the macroscopic stiffness matrix $C^{T}$ of composites in Equation (1) are expressed as functions of the elastic modulus and Poisson’s ratio:

(17) $$C^{T}=f\left(E_{1}^{T},E_{2}^{T},E_{3}^{T},G_{12}^{T},G_{13}^{T},G_{23}^{T},v_{12},v_{13},v_{23}\right)$$

When damage occurs, the stiffness coefficients decrease with the decreasing elastic modulus and Poisson’s ratio. The degraded stiffness coefficients are denoted by $C^{T,d}$:

(18) $$C^{T,d}=f\left(E_{1}^{T,d},E_{2}^{T,d},E_{3}^{T,d},G_{12}^{T,d},G_{13}^{T,d},G_{23}^{T,d},v_{12}^{T,d},v_{13}^{T,d},v_{23}^{T,d}\right)$$

Damage factors are generally used to explicitly describe the degree of stiffness coefficient reduction. The stiffness coefficients can be expressed as the product of the damage factor and their initial values at the current temperature. It should be noted that the damage factor is defined as a dimensionless relative reduction ratio, rather than an absolute stiffness value. The temperature dependence of absolute material properties has been fully incorporated into the initial undamaged modulus and strength via Equations (6), (7) and (10). Within the studied temperature range (−70 °C to 120 °C), the AC531 epoxy resin remains in the glassy state, with a glass transition temperature of approximately 230 °C. Although matrix ductility increases with rising temperature, which affects the damage initiation threshold and propagation rate, the relative residual stiffness after complete damage formation is dominated by the geometric nature of failure modes (e.g., through-thickness matrix cracks, fiber fracture) and remains relatively stable in the glassy region. Therefore, the assumption of temperature-independent degradation factors is a reasonable and widely adopted simplification in thermo-mechanical progressive damage analysis of composites.

Based on micromechanics, when constituent damage occurs in a ply of composite materials, the ply loses load-bearing capacity in certain directions while retaining partial load-bearing capacity in other directions. The stiffness degradation factors adopted in this work are determined with reference to classical progressive damage models for unidirectional carbon fiber–epoxy composites and are consistent with widely validated values for similar material systems [38]. After damage initiation, the correspondence between the stiffness reduction factors of the macroscopic equivalent stiffness of the unidirectional ply and the damage failure states is presented in Table 3.

#### 2.4.5. Analysis Methods for Macroscopic Properties of Composite Lamina

The fundamental mechanics-based rule of mixtures is widely used to calculate the macroscopic mechanical properties and CTE of composite laminates. For elastic properties, the VSPK micromechanical model proposed by Vignoli et al. [39,40,41,42] is adopted in this study. Taking into account the temperature dependence of material properties, the following equations are employed for estimation:

(19) $$E_{1}^{T}=V_{f}E_{1,f}+\left(1-V_{f}\right)E_{m}^{T}$$

(20) $$v_{12}=v_{13}=V_{f}v_{12,f}+\left(1-V_{f}\right)v_{m}$$

(21) $$v_{23}=V_{f}v_{23,f}+\left(1-V_{f}\right)v_{m}$$

(22) $$E_{2}^{T}=E_{3}^{T}=E_{m}^{T}\left(\frac{1}{1+\xi_{E_{2}}\left[\left(E_{m}^{T}/E_{2,f}\right)-1\right]V_{f}}\right)$$

(23) $$G_{12}^{T}=G_{13}^{T}=G_{m}^{T}\left(\frac{1}{1+\xi_{G_{12}}\left[\left(G_{m}^{T}/G_{12,f}\right)-1\right]V_{f}}\right)$$

(24) $$G_{23}^{T}=G_{m}^{T}\left(\frac{1}{1+\xi_{G_{23}}\left[\left(G_{m}^{T}/G_{23,f}\right)-1\right]V_{f}}\right)$$

where $G_{m}^{T}=E_{m}^{T}/2(1+\nu_{m})$ is the shear modulus of the matrix at temperature T, and $\xi_{E_{2}}$, $\xi_{G_{12}}$ and $\xi_{G_{23}}$ are calibration functions defined as follows:

(25) $$\xi_{E_{2}}=\left[c_{1}+c_{2}V_{f}+c_{3}\left(E_{m}^{T}/E_{2,f}\right)\right]$$

(26) $$\xi_{G_{12}}=\left[c_{4}+c_{5}V_{f}+c_{6}\left(G_{m}^{T}/G_{12,f}\right)\right]$$

(27) $$\xi_{G_{23}}=\left[c_{7}+c_{8}V_{f}+c_{9}\left(G_{m}^{T}/G_{23,f}\right)\right]$$

where $c_{i}$ are calibrated using experimental data. The calibration parameters used in this study are listed below [39]:

a1 = 2.0930, a2 = −1.4359, a3 = 0.0059;

a4 = 2.3145, a5 = −1.6043, a6 = −0.4199;

a7 = 1.7906, a8 = −0.9657, a9 = 0.0065.

For CTE, it can be expressed by the modified rule of mixtures [43,44]:

(28) $$\alpha_{1}=\frac{\alpha_{1,f}E_{1,f}V_{f}+\alpha_{m}E_{m}^{T}V_{m}}{E_{1,f}V_{f}+E_{m}^{T}V_{m}}$$

(29) $$\alpha_{2}=\alpha_{3}=\alpha_{2,f}V_{f}+\alpha_{m}(1-V_{f})+\left(\frac{E_{f1}v_{m}-E_{m}v_{21,f}}{E_{1}^{T}}\right)\left(\alpha_{m}-\alpha_{1,f}\right)V_{f}(1-V_{f})$$

### 2.5. Metallic Elastoplasticity

Under thermal environments, the CTEs of metallic materials are much larger than those of composite laminates. In mechanical joints, the hybrid-lap-jointed metallic material plates and composite laminates lead to the generation of thermal stresses inside the structure due to the difference in their CTEs. Therefore, in the analysis, to improve the accuracy of the simulation, the metallic elastoplastic model is adopted, and the CTE of the metal is incorporated.

### 2.6. Model Implementation

**Figure 5:**
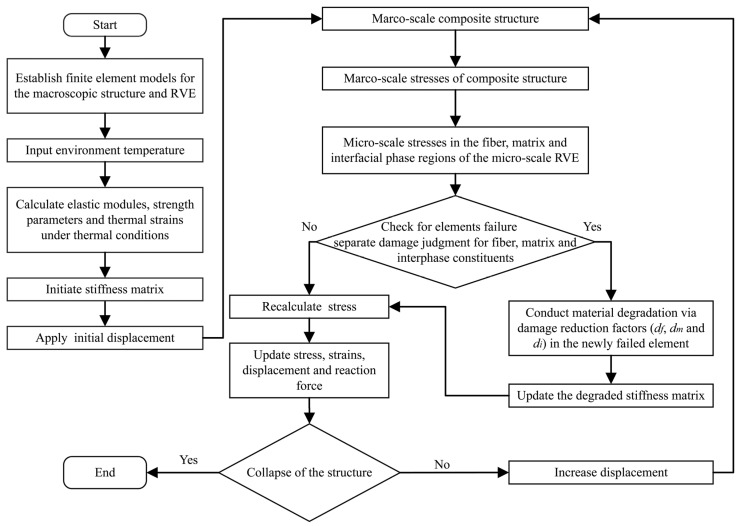
Flowchart of continuum damage model under thermal effects.

In the general-purpose finite-element software ABAQUS^®^ 6.14, the aforementioned failure criteria and material degradation model are incorporated into the user-defined subroutine UMAT, which enables the nonlinear progressive failure analysis of the metal–composite hybrid mechanical joint structure under different temperatures. The flowchart of the proposed model is shown in Figure 5, and its main steps are as follows:

Step 1: Through the observation of the micro-morphological characteristics of the composite, establish the finite-element model of the RVE. Based on the material properties of the three constituent phases (carbon fibers, resin matrix, and interfacial phase), predict the equivalent stiffness properties of the composite, as well as the mesoscopic stress field under per unit macroscopic stress/temperature;
Step 2: Establish the finite-element model of the macroscopic composite structure and adopt the equivalent property parameters predicted in Step 1 as the material properties of this macroscopic model. Apply the initial load and obtain the macroscopic strain of the composite structure through finite-element calculation and analysis;
Step 3: Superimpose the macroscopic stress on the stress field of the mesoscopic model in the form of a superposition coefficient to obtain the mesoscopic stress field of the macroscopic structure. Input the mesoscopic stress into the corresponding mesoscopic failure criteria to determine whether damage occurs in the composite structure and perform stiffness reduction on the damaged elements;
Step 4: Gradually increase the load through fixed incremental steps and continue to analyze the structure until the structural failure load is reached.

## 3. Experimental Procedures and Numerical Models

To verify the accuracy of the model predictions, static tensile tests were conducted on composite–aluminum alloy three-bolt double-shear joint specimens under three temperature conditions: LT, RT and HT. Meanwhile, a finite-element model of the joint structure was established using ABAQUS software. The simulation results were compared with the experimental results to validate the prediction accuracy of the proposed model.

### 3.1. Specimens

**Figure 6:**
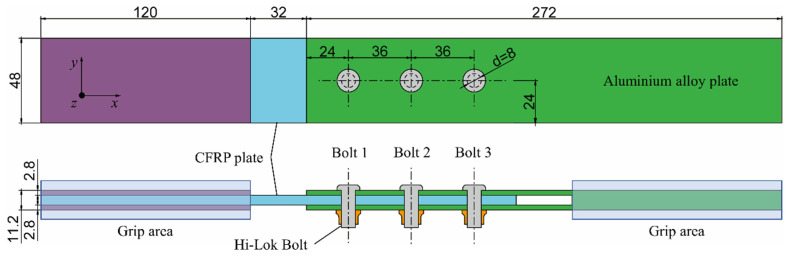
Test specimen geometric dimensions.

The specimen is a CFRP composite–aluminum alloy three-bolt double-shear joint, with dimensions shown in Figure 6. The composite laminate is fabricated from AC531/CCF800H composite material, with a layup sequence of [45/0/−45/90/0/0/45/0/−45/0]_2s_ and a nominal thickness of *t_C_* = 5.6 mm. The unidirectional tape is an orthotropic material, with a single-ply thickness of *t_lami_* = 0.14 mm.

The metal plates are made of 7075-T7451 aluminum alloy, with a thickness of *t_Alum_* = 5.6 mm, an elastic modulus of 74.5 GPa, a Poisson’s ratio of 0.3, and a CTE of 2.25 × 10^−5^/°C. Titanium alloy bolts are used, with a diameter D of 8 mm. The bolts are fabricated from Ti-6Al-4V titanium alloy, with an elastic modulus of 112 GPa, a Poisson’s ratio of 0.34, a yield strength of 909 MPa, and a CTE of 9.1 × 10^−6^/°C. The dimensions of the specimen are shown in Figure 6. Both lap plates have a length of 272 mm. For the joint, the pitch-to-diameter ratio is p/D = 4.5, the edge distance-to-diameter ratio is S_w_/D = 3, and the end distance-to-diameter ratio is e/D = 3. These geometric ratios are designed to ensure bearing failure of the joint, in accordance with the BOJCAS [45] (Bolted Joints in Composite Aircraft Structures) standard.

### 3.2. Static Strength Tests

**Figure 7:**
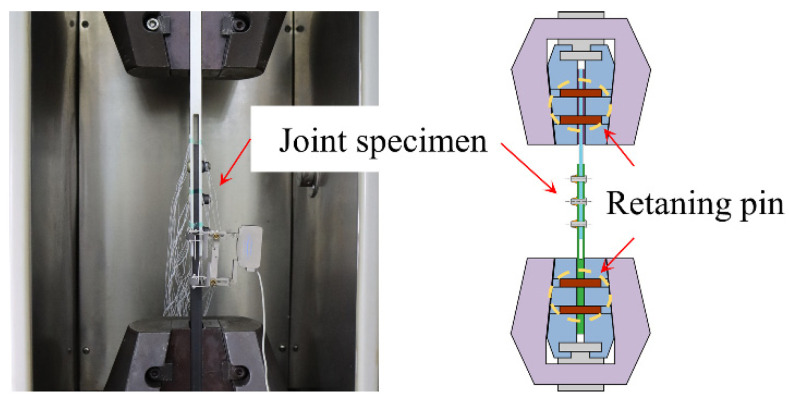
Tensile test of joints in universal testing machine with temperature chamber.

According to the ASTM D5961/D5961M [46] test standard, tensile tests were conducted on an electronic universal testing machine. The load was applied at a constant crosshead displacement rate of 2 mm/min until the specimen experienced complete failure or a sudden drop in the load curve occurred. During the tests, the environmental temperature was controlled by a high–low temperature environmental chamber, where the high-temperature and low-temperature environments were achieved using high-temperature alloy heating tubes and a refrigeration compressor, respectively. Strain data and load–displacement data were continuously collected by a digital data acquisition system. The peak value of the load–displacement curve was taken as the ultimate failure load, and the failure mode of the specimen was recorded by photographs. The experimental setup is shown in Figure 7.

### 3.3. Progressive Failure Analysis Models of Specimens

**Figure 8:**
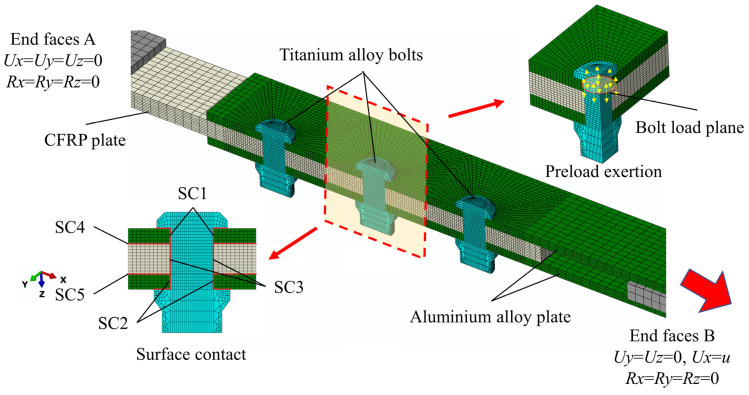
Finite-element model of composite-metal hybrid joints.

A full three-dimensional (3D) finite-element model of the specimen was built using ABAQUS^®^ software for accurate stress analysis and damage evolution analysis. For clarity, Figure 8 shows the x-z plane cross-section of the model with boundary conditions.

In the numerical model, aerospace high-lock bolts and nuts were merged into a single dumbbell-shaped part. This simplifies extra contact surfaces and improves calculation efficiency. For composite laminates, C3D8R eight-node solid elements were used. One element was assigned per four layers in the through-thickness direction. For metal plates, five elements were used in the through-thickness direction.

A total of 84 seeds were evenly placed around the holes in the connecting plates. This ensures uniform mesh distribution on the contact surfaces between the lap plates and the bolts near the holes. To eliminate the influence of mesh density on the prediction of stress concentration and damage evolution around bolt holes, a mesh convergence study was conducted with reference to the classical evaluation framework for composite progressive damage analysis proposed by Zhang et al. [5]. Three mesh schemes with progressive refinement around the bolt hole periphery were designed, with approximately 40, 80 and 120 elements distributed circumferentially around each bolt hole per ply, corresponding to coarse, medium and fine mesh densities.

The ultimate tensile load of the hybrid joint was selected as the sole convergence evaluation metric. The results indicate that, when the mesh is refined from the medium scheme to the fine scheme, the relative deviation of the predicted ultimate tensile load is only 1.8%. This deviation falls below the standard 3% convergence threshold, demonstrating that further mesh refinement barely changes the predicted bearing capacity of the joint. The medium mesh scheme was ultimately adopted for all numerical simulations to balance computational accuracy and efficiency.

The friction coefficient between the bolts and connecting plates was set to 0.1, and the friction coefficient between the connecting plates was set to 0.2 [24]. All degrees of freedom (DOFs) of end face A on the aluminum plate were fully constrained. On end face B of the composite laminate, all DOFs except x-direction displacement were constrained. A tensile displacement load was applied in the x-direction to simulate the static tensile process.

Simulation at RT included two steps: (1) applying the bolt preload and (2) applying the displacement load to simulate static tension. Simulation at HT/LT temperature conditions included three steps: (1) applying the bolt preload; (2) simulating the thermal process of the structure: heating from 20 °C to 120 °C or cooling from 20 °C to −70 °C; and (3) simulating the static tensile process. The analysis steps were coupled with the UMAT subroutine to simulate progressive damage in the composites. For the metallic parts, plasticity parameters were defined to consider the effect of metal plastic deformation on the joint structure.

In the finite-element analysis, temperature effects are introduced in two ways: The effect of temperature on the stress–strain relationship of composites is achieved by inputting temperature and thermal expansion coefficients into the user-developed UMAT subroutine. The elastic modulus and strength of the epoxy resin matrix are calculated by Equations (6) and (7). The elastic modulus and strength of the interphase are calculated by Equation (10). The macroscopic mechanical properties of composite laminates are calculated using the rule of mixtures in Section 2.4.5.

In addition, the failure analysis model is established using the failure criteria in Section 2.4 and the material degradation model in Table 3. The stiffness reduction factors adopted in this work are from the classic progressive damage framework proposed by Camanho and Matthews [38], which has been extensively validated and widely applied in carbon fiber-reinforced epoxy composite systems. This set of degradation factors characterizes the residual load-bearing capacity of unidirectional plies corresponding to different failure modes, which is governed by the universal physical mechanism of composite damage: fiber tensile fracture almost completely eliminates the axial load-bearing capacity, while matrix cracking retains partial transverse and shear stiffness. Since the AC531/CCF800 system belongs to the typical thermoset carbon fiber–epoxy composite family and shares consistent failure modes with the reference system, the adoption of these classic degradation factors is physically justified. A sensitivity analysis is provided in Section 4 to further verify the robustness of the results. The stiffness reduction factors are set as follows: $d_{f\_t}$ = 0.01, $d_{f\_c}$ = 0.2, $d_{m\_1}$ = 0.4, $d_{m\_2}$ = 0.2, $d_{i\_1}$ = 0.4, $d_{i\_2}$ = 0.2.

## 4. Results and Discussion

The failure behavior of three-bolt double-shear joints between composite and aluminum alloy lap plates under LT, RT and HT conditions is predicted using the proposed continuous damage model. A comparative analysis is conducted between the experimental and numerical simulation results from four aspects: ultimate load-bearing capacity, load–displacement curves, damage evolution laws and failure modes. This verifies the validity of the proposed model and reveals the influence mechanism of thermal effects on the mechanical properties of hybrid joint structures.

Five tensile tests were conducted for each group under the three temperature conditions. The average value of the ultimate failure loads was taken as the experimental result. Meanwhile, the failure behavior of each group of specimens was predicted through progressive failure analysis. Table 4 presents the failure loads, average failure load ($\bar{x}$), standard deviation (SD), coefficient of variation (CV) and finite-element simulation results for all specimens.

It can be seen from Table 4 that the standard deviations under all temperature conditions are less than 3 kN, and the coefficients of variation are all less than 3%. This indicates that the experimental results have good repeatability and stability. Under LT, RT and HT conditions, the relative errors between the numerical predictions and the experimental mean values are 2.83%, 0.79% and 1.27%, respectively, all of which are less than 3%. More importantly, all predicted ultimate loads fall within the experimental scatter band defined by the mean value ± one standard deviation, verifying that the model predictions are well within the natural dispersion range of the test data. The above results fully demonstrate that the established multiscale progressive failure model can accurately and reliably predict the ultimate load-bearing capacity of composite–metal hybrid bolted joint structures under thermo-mechanical coupled loads.

**Figure 9:**
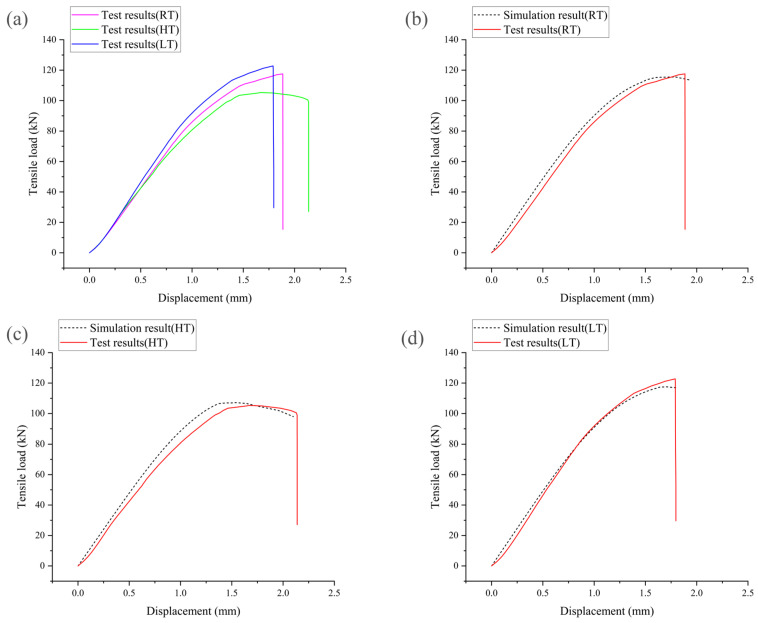
Typical load–displacement curves. (**a**) Test results under RT, HT and LT conditions; (**b**) test results and simulation result under RT; (**c**) test results and simulation result under HT; (**d**) Test results and simulation result under LT.

Figure 9 presents the comparison of experimental load–displacement curves for composite–aluminum alloy bolted joint structures under LT, RT and HT conditions, as well as the comparison between the experimental and numerical simulation results at each temperature. The bolt hole numbering is consistent with that in Figure 6. The load-displacement curves at different temperatures all exhibit typical elastoplastic response characteristics. In the initial loading stage, the load increases approximately linearly with the displacement, and the structure is in the elastic deformation stage. As the displacement increases further, the curve gradually deviates from linearity and enters the nonlinear deformation stage. When the load reaches the ultimate value, the curve drops rapidly, and the structure undergoes final failure.

To quantitatively validate the model performance throughout the full loading process, three key metrics—initial stiffness, displacement at ultimate load, and onset of nonlinearity—are extracted from the curves for systematic comparison. The initial stiffness is defined as the slope of the linear elastic segment. The relative errors between numerical predictions and experimental values are 2.1%, 2.0% and 2.6% at LT, RT and HT conditions, respectively, all within 3%, verifying that the model accurately captures the linear elastic deformation of the joint. The displacement corresponding to the ultimate load is adopted as the failure displacement indicator, with prediction errors of 2.9%, 2.8% and 3.9% at the three temperatures, all below 4%. The onset of nonlinearity is defined as the load level at which the curve deviates from the linear trend by 5%, marking the initiation of remarkable matrix damage and stiffness degradation. The predicted nonlinear onset loads deviate from the experimental values by 2.6%, 2.6% and 3.1% at LT, RT and HT, respectively. As the temperature rises, the nonlinear stage initiates earlier, which is attributed to the softening of the epoxy resin matrix that accelerates early damage initiation.

Overall, the simulation curves at all temperatures show good agreement with the experimental curves in terms of overall trend, ultimate load, stiffness evolution and failure deformation characteristics. This indicates that the established multiscale progressive failure model can accurately describe the full-process mechanical response of hybrid joint structures under thermo-mechanical coupled loads and provides a reliable numerical basis for subsequent damage evolution analysis.

In addition, it can be seen from Figure 9a that the ultimate load-bearing capacity of the joint structure exhibits significant temperature dependence. The ultimate load is the highest under LT conditions, followed by RT, and the lowest under HT conditions. Meanwhile, the curve deviates from the linear stage earlier under HT conditions. This indicates that the softening of the epoxy resin matrix leads to premature stiffness degradation of the structure. In contrast, the linear segment of the curve is significantly longer under LT conditions. This reflects the enhancement effect of the matrix low-temperature hardening effect on the overall stiffness and load-bearing performance of the structure. Quantitative comparison results show that the simulation curve almost completely coincides with the experimental curve at RT. The prediction errors of both ultimate load and initial stiffness are less than 2%. Under HT conditions, the peak occurrence time of the simulation curve is slightly earlier than that of the experimental curve. However, the overall trend and nonlinear development characteristics are highly consistent. The model can accurately reflect the influence law of resin matrix softening and interfacial debonding on the structural mechanical response. Under LT conditions, the simulation and experimental curves also show good agreement. The prediction error of ultimate load is less than 4%. This verifies the accurate description capability of the established model for the low-temperature strengthening effect of composites.

Specifically, the experimental ultimate load of the joint structure increases by 3.91% under LT conditions, compared with RT conditions. The enhancement mechanism is as follows. Under LT conditions, the AC531 epoxy resin matrix is in a high-modulus glassy state. The thermal motion of molecular segments is significantly inhibited, resulting in low-temperature hardening. Both its elastic modulus and strength are significantly improved. Meanwhile, the interfacial bonding strength between the carbon fibers and the epoxy resin matrix is also enhanced at LT conditions. This effectively suppresses the initiation and propagation of interfacial debonding damage, which improves the load-bearing capacity of the composite laminate and thus increases the ultimate load of the entire joint structure.

In contrast, the experimental ultimate load of the joint structure decreases by 9.07% under HT conditions compared with RT conditions. When the temperature reaches 120 °C, the resin matrix is highly softened. According to Equation (5), its elastic modulus decreases by 23%. Meanwhile, calculated by Equation (7), its tensile strength decreases by 12% at 120 °C. In addition, the difference in thermal expansion coefficients between the fibers and the matrix induces large thermal stresses at the interface. This accelerates interfacial debonding failure, causes premature damage in the composite laminate, and thus significantly reduces the ultimate load-bearing capacity of the entire joint structure.

**Figure 10:**
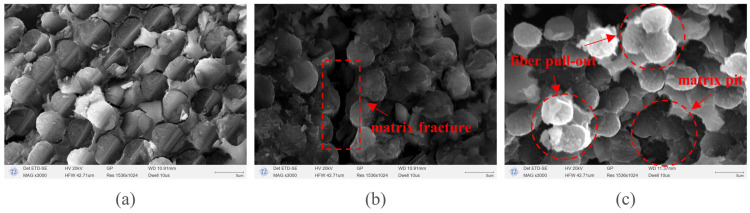
SEM micrographs of tensile fracture surfaces of composites under different temperature conditions: (**a**) LT conditions; (**b**) RT conditions; (**c**) HT conditions.

SEM was used to analyze the micro-morphology of damaged CFRP composites at different temperatures. The typical morphologies are shown in Figure 10. Under LT conditions, the composite exhibits relatively flat fracture characteristics. The fibers are neatly aligned along the loading direction, and there is no obvious interfacial separation between the matrix and fibers. This micro-morphology feature directly reflects that strong interfacial bonding is formed between carbon fibers and epoxy resin matrix at LT conditions. This bonding can effectively transfer loads and fully exert the reinforcing effect of carbon fibers.

Under RT conditions, the fracture morphology of the composite shows obvious “brittle-ductile” mixed fracture characteristics. Compared with the low-temperature fracture, the flatness of the room-temperature fracture decreases, and a certain number of short fiber pull-outs and corresponding matrix pits appear. This indicates that the toughness of the epoxy resin matrix is improved at RT. During the tensile process, in addition to matrix brittle fracture and fiber fracture, interfacial debonding also occurs.

Under HT conditions, the fracture morphology of the composite shows typical ductile fracture characteristics. A large number of long fiber pull-outs and exposed fiber surfaces can be observed, and there are obvious interfacial separation gaps between the fibers and the matrix. The fiber pull-out length is significantly longer than that at RT and LT, and the surfaces of the pulled-out fibers are smooth, with almost no resin residue.

The above results indicate that the modulus and strength of the epoxy resin matrix decrease under HT conditions. Meanwhile, the interfacial bonding force between the carbon fibers and the matrix is significantly weakened, making the interface the weak link of the composite. Under tensile load, interfacial debonding failure occurs prematurely, which cannot effectively transfer loads. This leads to the inability of the reinforcing fibers to bear loads cooperatively, thus reducing the load-bearing capacity of CFRP composites.

**Figure 11:**
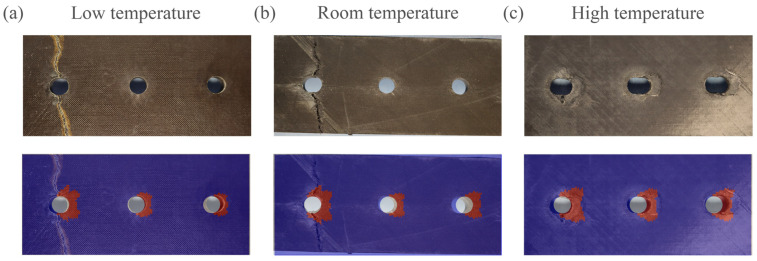
Failure modes of composites under different temperature conditions: (**a**) LT conditions; (**b**) RT conditions; (**c**) HT conditions.

Since the load-bearing failure of composite–aluminum alloy bolted joint structures is mainly dominated by the damage evolution and propagation in the area around the composite holes, the composite hole periphery is selected for analysis. In this study, macroscopic morphology observation and numerical simulation analysis were conducted on the composite hole periphery of the failed joint structures. The comparison between experimental failure morphologies and finite-element simulation damage contours of composites in the joint structures under different temperature conditions is shown in Figure 11. Under LT and RT conditions, the main failure mode is through-thickness cracks propagating from the hole periphery to the plate end. The bearing damage area around the hole is small at LT and increases at RT. When the temperature reaches 120 °C, no obvious through-thickness cracks are observed. The main failure mode is large-area bearing damage around the hole, and the damage area is significantly expanded.

**Figure 12:**
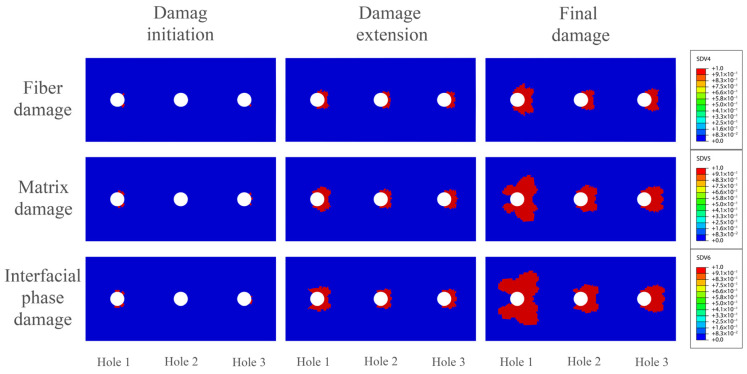
Damage evolution process of composite hole periphery at RT.

**Figure 13:**
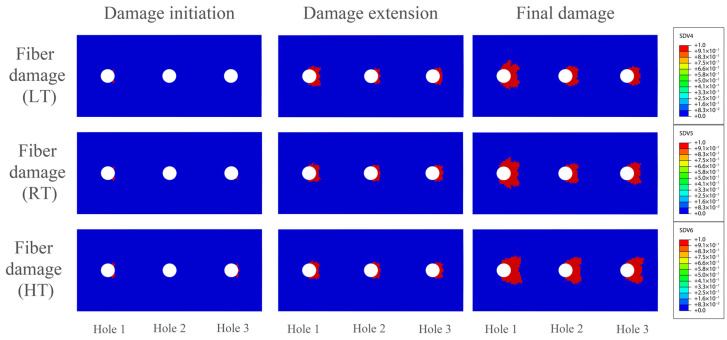
Fiber damage evolution process of composite hole periphery under different temperature conditions.

All damage contours presented in Figure 11, Figure 12 and Figure 13 are extracted as element-level state variables from the user-defined UMAT subroutine. The three damage modes of fiber, matrix and interphase are independently judged at each integration point according to the corresponding mesoscopic failure criteria established in Section 2.4. The value of each damage variable reflects the stiffness degradation degree of the corresponding constituent after failure initiation: a value of zero represents the intact, undamaged state, while a value approaching one indicates that the corresponding failure criterion has been satisfied and the stiffness has been degraded according to the rules in Table 3. Three independent output variables are defined in the UMAT subroutine for fiber, matrix, and interphase damage, respectively, enabling separate visualization of the damage evolution process for each failure mode.

Figure 12 shows the evolution process contours of three damage modes (fiber, matrix and interphase) in carbon fiber–epoxy composite laminates at RT. Due to the obvious uneven load distribution in multi-bolt joint structures, the load proportion borne by the bolts at both ends is significantly higher than that borne by the middle bolt. At the damage initiation stage, damage first occurs on the bolt-bearing sides of Hole 1 and Hole 3 at both ends of the structure. No damage failure points appear around Hole 2. After entering the damage propagation stage, the damage areas of the matrix and interphase expand rapidly with the increasing load. The propagation rate of fiber damage is relatively slow. At this time, damage failure areas of different degrees appear around all bolt holes. However, the damage severity around Hole 1 is the most serious. When the load approaches the ultimate load-bearing capacity of the structure, the damage distribution law around the holes is basically consistent with that in the damage propagation stage. The damage around Hole 1 remains the most serious. At this point, the stress concentration degree around Hole 1 exceeds the in-plane tensile strength of the composite. In addition to the bearing damage failure around the hole, tensile fracture damage initiating and propagating outward along the plate width direction begins to appear. This leads to the final tensile fracture failure of the composite, which is also consistent with the failure mode in the experiment.

To systematically reveal the effect of temperature on the damage evolution laws around carbon fiber–epoxy composite holes, Figure 13 presents the full-process evolution contours of fiber damage around composite holes under LT, RT and HT conditions. At the damage initiation stage, the damage initiation characteristics at different temperatures exhibit significant differences. Under LT conditions, local fiber damage only appears on the bolt-bearing side of Hole 1 at the structural end. Under RT conditions, fiber damage initiates simultaneously on the bearing sides of Hole 1 and Hole 3. The damage severity of Hole 1 is slightly higher than that of Hole 3. Under HT conditions, although damage still occurs simultaneously on the bearing sides of Hole 1 and Hole 3, the damage area and severity of the two holes are basically equivalent.

After entering the damage propagation stage, the aforementioned temperature-dependent characteristics become more prominent. Under LT and RT conditions, the damage around the holes always maintains a non-uniform distribution law. Hole 1 is the most severely damaged, followed by Hole 3, and Hole 2 is the least damaged. This distribution heterogeneity is more significant under LT conditions. In sharp contrast, the damage propagation rates and final damage severities of Hole 1 and Hole 3 are highly similar under HT conditions. No obvious damage concentration phenomenon is observed.

The aforementioned temperature dependence of damage distribution essentially originates from the significant thermal expansion coefficient mismatch between aluminum alloy bolts and CFRP composites. Under LT conditions, the shrinkage deformation of aluminum alloy is much larger than that of composites. This causes the local thermal stress around Hole 1 to be in the same direction as the bearing stress generated by mechanical loading, while the thermal stress around Hole 3 is opposite to the bearing stress. This stress superposition effect makes the actual load borne by Hole 1 significantly higher than that by other holes, so the damage is the most severe. Conversely, under HT conditions, the expansion deformation of aluminum alloy is larger than that of composites. This causes the local thermal stress around Hole 1 to be opposite to the bearing stress, while the thermal stress around Hole 3 is in the same direction as the bearing stress. This stress cancellation effect transfers part of the load originally borne by Hole 1 to Hole 3, which finally leads to the damage severities of the two holes tending to be consistent.

At the final damage stage, the failure modes at different temperatures also undergo a fundamental transformation. Under LT and RT conditions, Hole 1 always bears the highest load. A tensile fracture damage zone propagating along the plate width direction first initiates around Hole 1. When the load reaches the ultimate value, the structure undergoes sudden tensile fracture failure. Under HT conditions, large-area bearing damage appears around all bolt holes. The structure finally fails in the bolt hole bearing mode. This is because the increase in temperature leads to a significant decrease in the modulus and strength of the epoxy resin matrix, while the toughness of the composite is improved. This makes the hole’s periphery more prone to matrix shear deformation and fiber compressive buckling rather than brittle tensile fracture. The development of bolt hole bearing damage can effectively alleviate the inherent uneven bolt load distribution in multi-bolt joint structures. This ultimately causes almost simultaneous bearing failure at all hole positions.

## 5. Conclusions

This study establishes a temperature-dependent multi-scale progressive failure model based on micromechanical failure theory and systematically investigates the tensile mechanical behavior and failure mechanisms of AC531/CCF800H composite–7075 aluminum alloy three-bolt double-shear joints under LT (−70 °C), RT (20 °C), and HT (120 °C) conditions. The main conclusions are summarized as follows:

(1) The proposed multi-scale framework achieves cross-scale failure prediction from mesoscopic constituent damage to macroscopic structural response via macro–meso stress transformation, temperature-dependent property correction, mesoscopic constituent-specific failure criteria, and matched stiffness degradation rules. Compared with traditional macroscopic thermo-mechanical failure models that can only characterize the overall structural response, the proposed approach enables independent damage assessment for the fiber, matrix, and interphase phases and can distinguish their respective contributions to structural failure. Meanwhile, the framework avoids the prohibitive computational cost of full-scale refined mesoscopic modeling, striking a favorable balance between prediction accuracy and computational efficiency. The ultimate load prediction errors under all temperature conditions are below 5%, and the simulation results show good agreement with the experimental data in load–displacement curves, damage evolution paths, and failure modes, verifying the accuracy and engineering applicability of the model;
(2) Temperature significantly regulates the load-bearing performance of the structure. LT hardens the resin matrix and enhances interfacial bonding, which inhibits the initiation and propagation of damage. The ultimate load increases by 3.91% compared with RT. HT reduces the modulus and strength of the resin and aggravates interfacial debonding. Thermal expansion mismatch generates additional thermal stresses, resulting in a 9.07% decrease in ultimate load compared with RT;
(3) Temperature changes the bolt load distribution and damage evolution path. Under LT/RT conditions, the superposition of thermal and mechanical stresses leads to load concentration at the end of Hole 1 with the most severe damage, and the structure mainly fails by tensile fracture around the hole. Under HT conditions, thermal stresses offset the uneven load distribution. The damage at each hole tends to be consistent, and the structure mainly fails by full-hole periphery bearing failure;
(4) The mesoscopic failure mechanisms exhibit obvious temperature dependence. The fracture surface is flat with strong interfacial bonding at LT, and the failure is dominated by brittle fracture of fibers and matrix. RT shows a brittle–ductile mixed fracture accompanied by a small amount of fiber pull-out. At HT, severe interfacial debonding and a large number of long fiber pull-outs are observed, presenting typical ductile fracture characteristics. The interface and matrix are the weak links of the structure at HT.

From an engineering application perspective, the proposed multi-scale model provides an efficient and accurate numerical tool for thermo-mechanical strength verification and damage mechanism analysis of composite–metal hybrid bolted joints in aerospace service environments. It can support the structural design and parameter optimization of hybrid connection structures under extreme thermal conditions and reduce the dependence on large numbers of costly environmental mechanical tests in the design phase.

## Figures and Tables

**Table 1:** Material properties of carbon fiber.

Material Properties	CCF800H
$E_{1,f}$ (GPa)	252
$E_{2,f}$ = $E_{3,f}$ (GPa)	19
$G_{12,f}$ = $G_{13,f}$ (GPa)	27
$G_{23,f}$ (GPa)	7
$v_{23,f}$	0.33
$v_{12,f}$ = $v_{13,f}$	0.2
$\alpha_{11,f}$ (10^−6^/K)	−0.1
$\alpha_{22,f}$ = $\alpha_{33,f}$ (10^−6^/K)	6
$X_{t,f}$ (GPa)	5.47
$X_{c,f}$ (GPa)	3.54

**Table 2:** Material properties of matrix at RT.

Material Properties	AC531
$E_{m}$ (GPa)	3.5
$v_{m}$	0.33
$\alpha_{m}$ (10^−6^/K)	35.36
$X_{t,m}$ (MPa)	85
$X_{c,m}$ (MPa)	125

**Table 3:** Material degradation model for the failure criterion.

Failure Mode	Degradation Factors								
	$E_{1}^{T}$	$E_{2}^{T}$	$E_{3}^{T}$	$G_{12}^{T}$	$G_{13}^{T}$	$G_{23}^{T}$	$v_{12}$	$v_{13}$	$v_{23}$
Fiber tensile failure	$d_{f\_t}$	1	1	$d_{f\_t}$	$d_{f\_t}$	1	$d_{f\_t}$	$d_{f\_t}$	1
Fiber compression failure	$d_{f\_c}$	1	1	$d_{f\_c}$	$d_{f\_c}$	1	$d_{f\_c}$	$d_{f\_c}$	1
Matrix failure	1	$d_{m\_1}$	$d_{m\_1}$	$d_{m\_1}$	$d_{m\_1}$	$d_{m\_2}$	$d_{m\_1}$	$d_{m\_1}$	$d_{m\_2}$
Interphase failure	1	$d_{i\_1}$	$d_{i\_1}$	$d_{i\_1}$	$d_{i\_1}$	$d_{i\_2}$	$d_{i\_1}$	$d_{i\_1}$	$d_{i\_2}$

**Table 4:** Comparison of experimental and numerical ultimate loads of the hybrid bolted joint at different temperatures.

No.	Environment	Failure Load/kN	$\bar{x}$/kN	SD/kN	CV/%	Numerical Result/kN	Error/%
LT-1	−70	119.81	120.95	2.99	2.47	117.53	2.83
LT-2		122.82					
LT-3		123.51					
LT-4		122.37					
LT-5		116.22					
RT-1	23	117.69	116.40	1.24	1.07	115.48	0.79
RT-2		115.28					
RT-3		117.56					
RT-4		116.45					
RT-5		115.02					
HT-1	120	105.33	105.85	0.85	0.80	107.19	1.27
HT-2		105.78					
HT-3		106.01					
HT-4		104.94					
HT-5		107.18					

## Data Availability

The original contributions presented in this study are included in the article. Further inquiries can be directed to the corresponding author.
